# A Method for the Evaluation of Image Quality According to the Recognition Effectiveness of Objects in the Optical Remote Sensing Image Using Machine Learning Algorithm

**DOI:** 10.1371/journal.pone.0086528

**Published:** 2014-01-28

**Authors:** Tao Yuan, Xinqi Zheng, Xuan Hu, Wei Zhou, Wei Wang

**Affiliations:** 1 School of Land Sciences and Technology, China University of Geosciences, Beijing, China; 2 Key Laboratory of Land Regulation, Ministry of Land and Resources, Beijing, China; New York University, United States of America

## Abstract

Objective and effective image quality assessment (IQA) is directly related to the application of optical remote sensing images (ORSI). In this study, a new IQA method of standardizing the target object recognition rate (ORR) is presented to reflect quality. First, several quality degradation treatments with high-resolution ORSIs are implemented to model the ORSIs obtained in different imaging conditions; then, a machine learning algorithm is adopted for recognition experiments on a chosen target object to obtain ORRs; finally, a comparison with commonly used IQA indicators was performed to reveal their applicability and limitations. The results showed that the ORR of the original ORSI was calculated to be up to 81.95%, whereas the ORR ratios of the quality-degraded images to the original images were 65.52%, 64.58%, 71.21%, and 73.11%. The results show that these data can more accurately reflect the advantages and disadvantages of different images in object identification and information extraction when compared with conventional digital image assessment indexes. By recognizing the difference in image quality from the application effect perspective, using a machine learning algorithm to extract regional gray scale features of typical objects in the image for analysis, and quantitatively assessing quality of ORSI according to the difference, this method provides a new approach for objective ORSI assessment.

## Introduction

Currently, remote sensing image data, mainly optical remote sensing image (ORSI) data, are a major data source to obtain spatial information. ORSI quality is closely related to its application effects, and the difference in different-quality images during object identification and information extraction is very obvious. How to accurately describe and quantitatively express the quality difference is very important to ORSI applications.

At present, ORSI quality is assessed largely by two different types of methods. The first is the subjective assessment method; that is, the ORSI quality level is judged based on some pre-specified scale standards, or the experiences of the observers on the visual effects of the images. For this method, the National Imagery Interpretability Rating Scale (NIIRS) standard is still extensively applied in remote sensing applications. This standard correlates the task needs of the user with the image quality. The second is the objective assessment method including (1) error statistics, (2) human visual system (HVS) characteristics, and (3) structural similarity (SSIM). The error statistical method obtains the differences by comparing the distorted image with the reference image through the design features, finds a number of statistical quantities, and links them to the image quality. The HVS method utilizes the fact that human eyes are inherently insensitive to some image distortion and that the nature and distribution of different noises may cause significant differences in visual effects. This method is mainly performed by simulating human subjective feeling to establish physical model fitting for human visual perception. It often uses parameters such as the distortion sensitivity, edge distortion, and sharpness to analyze image quality. The error statistical method considers a digital image as a collection of isolated image pixels, ignores the statistical correlation among local pixels, and identifies signal errors in the visual perception leading to quality aberration, which do not conform to human visual characteristics. The HVS method has several advantages, but its channel decomposition algorithm is too complex and does not sufficiently consider the correlation among pixels, leading to great differences from the real visual perception. Therefore, in certain test conditions and if there are different image distortions, its superiority over PSNR and MSE is not evident [1].

In short, image quality evaluation is an issue that has garnered worldwide interest and has made substantial progress. However, there are still many issues that should be investigated.

1. Although the subjective assessment method represented by NIIRS has several disadvantages, such as being time-consuming, having a high cost, and requiring multiple repetitions, it is still widely used in the remote sensing (RS) field because its assessment results are closely associated with RS application effects.
2. Objective assessment methods originating from the field of digital image or video signal processing rarely consider specific tasks and application effects of ORSI data. Commonly used assessment parameters are the variance, kurtosis, entropy, contrast, sharpness, average gradient, and edge intensity [2]–[3]. Based on current study results, the quantitative assessment indexes of image quality reflect some aspects of the ORSI quality under certain conditions. However, for these indicators of digital image processing, the calculation results are often significantly different from the practical application effects of object identification and information extraction of remote sensing data, or even completely opposite.
3. These objective assessment methods utilize full-reference image quality assessment; thus, their results are relative to original reference images [4]–[5]. However, reference images are not used in RS practical applications; thus, these methods have limited application value in the RS field.

After the above analysis, we find that ORSI differs from methods using a common digital image. ORSI is mainly used for object identification and information extraction, and its quality is mainly reflected in the capacity of object identification and information extraction. Therefore, an ORSI quality evaluation method must be able to reflect this difference, and the evaluation results must be consistent with practical application effects. The only evaluation method now in line with this requirement is NIIRS, which is a subjective evaluation. Starting from the practical application effects of ORSI data, this study will analyze the relationship between object identification effects and image quality, and further establish an objective and quantitative ORSI quality evaluation method.

In this study, with the focus on the practical application of ORSI data, we evaluated the image quality given the ability of optical remote sensing data to recognize target objects, revealed the relationship between the commonly used objective assessment indexes and the practical application effects of RS data, and analyzed the applicability and limitations of these indexes used to represent the image data quality.

On this basis, we used a machine learning algorithm, selected sample training classifiers, and automatically identified and labeled typical objects in ORSI by programming in the Microsoft Visual C++2008 and OpenCV 2.1 environment. We then quantified the recognition results and defined the recognition rate. By standardizing the recognition rate, the practical application effects of the different qualities of ORSI can be better reflected. We propose an application-oriented ORSI quality assessment method that provides a new perspective from which to assess ORSI quality.

## Materials and Methods

### (1) Data Source

WorldView-2 image data are one set of commercial satellite data whose spatial resolution of the panchromatic wave band is as good as 0.5 m. Thus, the Worldview-2 panchromatic wave band data, ortho-rectified in March 2010 by Zhengzhou City, Henan Province, China, were chosen as our experimental data to study the object recognition effect. A visual inspection shows that the data have clear texture without cloud coverage and striped dead pixels.

### (2) Data Pre-processing

**Figure 1:**
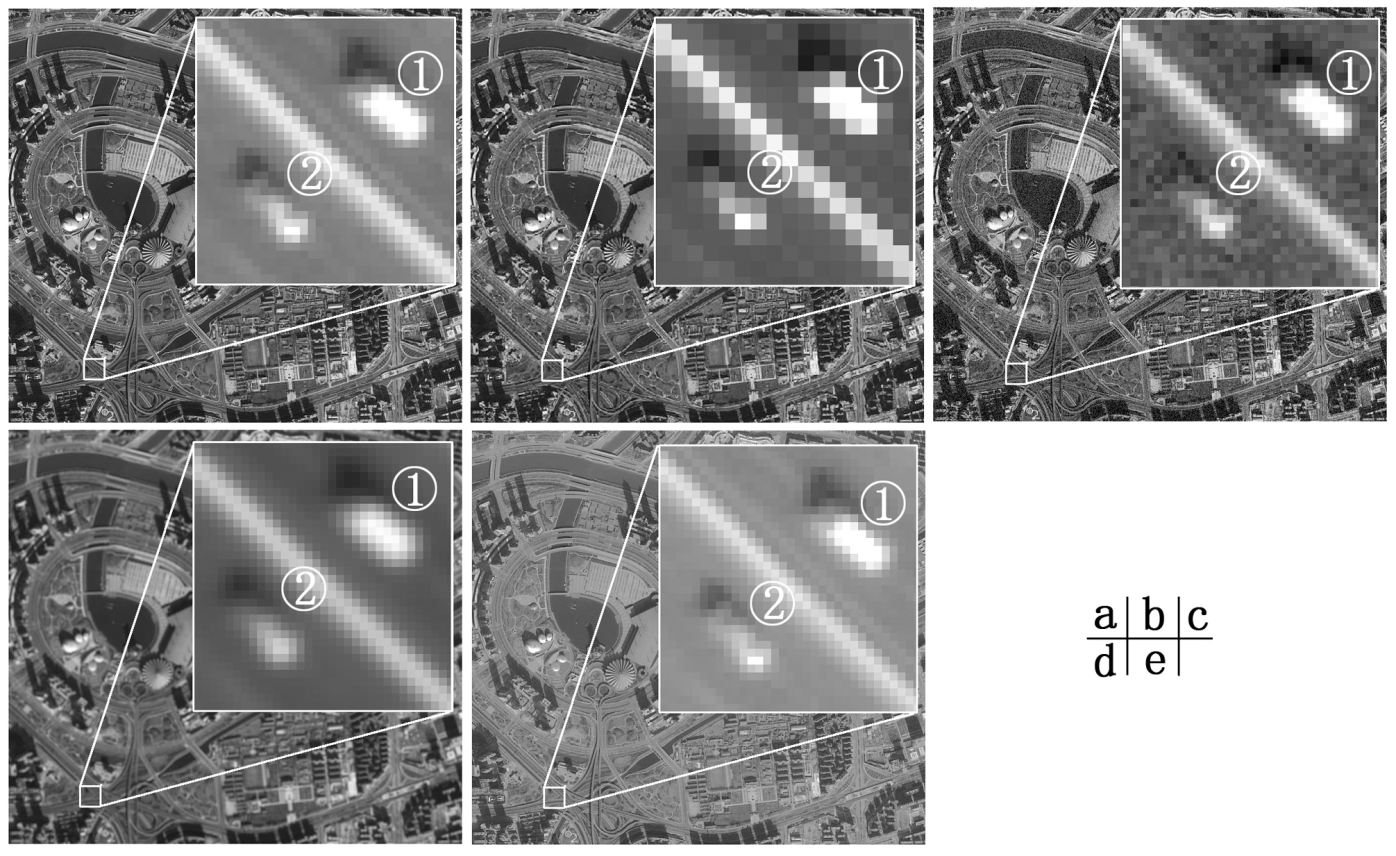
Comparison between the original image and the images after quality-reduction processing. (a) original image, (b) reduced-resolution image, (c) added Gaussian noise image, (d) defocus-blurred image, and (e) reduced contrast image.

To simulate the image data acquired by different sensors in different imaging conditions, the original image was re-acquired, its resolution was reduced, and it was used to model the image data obtained by different sensors. In order to model the image data acquired by the same sensor in different imaging conditions, three methods, including adding Gaussian noise, defocus blurring, and reducing image contrast, were used for image processing. The original image and images processed by using the above methods are shown in Fig. 1: (a) the original image, (b) a resolution-reduced image, (c) a Gaussian noise-added image, (d) a defocus-blurred image, and (e) a contrast-reduced image.

**Figure 2:**
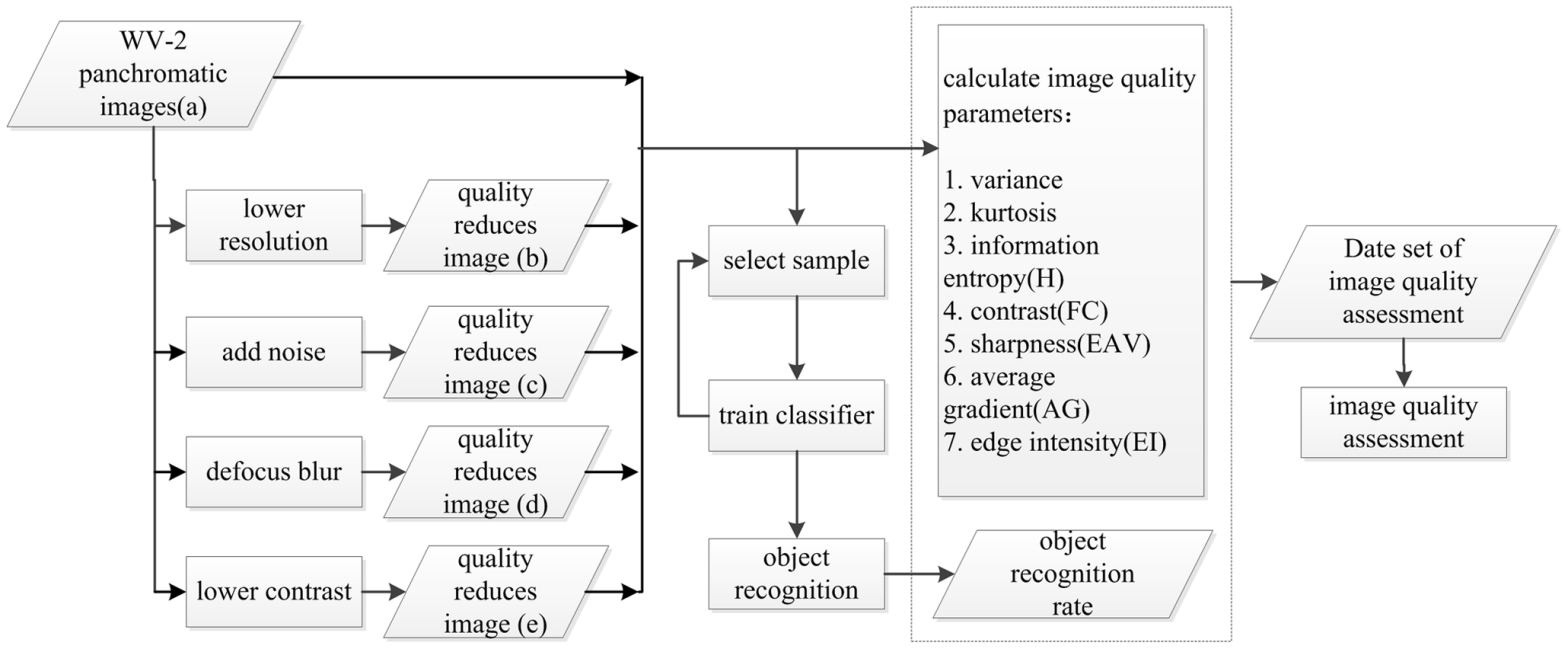
Flowchart of image quality assessment based on object recognition effect.

After the quality-reduction processing, we used a MATLAB program to calculate commonly used image quality indicators, such as the entropy, contrast, and sharpness, for the original image (a) and the other quality-reduced images (b), (c), (d), and (e). Then, we wrote a target recognition program based on the computer vision function library, OpenCV, selected positive and negative samples at the same positions in images (a), (b), (c), (d), and (e), utilized the Adaboost algorithm and Haar features of the target object, trained the target recognition classifier, obtained five classifiers numbered A, B, C, D, and E, respectively, and used them to examine the corresponding images (a), (b), (c), (d), and (e). After the testing program was executed, the program automatically marked the identified targets. Calculation of the ORRs of different images characterized the differences in image quality, and the image quality assessment method was presented by comparing the differences between the above identified effects and the commonly used image quality assessment parameters. The specific program execution flow is shown in Fig. 2.

### (3) Selection of Recognized Objects

Automobiles are commonly distributed in urban areas and relatively easy to distinguish from their surrounding objects. More importantly, they are of a size close to the object identification limit of ORSIs (meter and sub-meter size) and have simple but unique statistical structures in an image. Thus, using automobiles as recognized objects can best reveal the effect of the image quality on object recognition and is convenient for us to determine their overall statistical quantities and recognition rates in recognition experiments.

### (4) Selection of Object Recognition Methods

To compare the differences in the effects of image quality on object recognition, the methods used for object recognition should avoid human intervention and be sufficiently sensitive to the changes in image quality. The commonly used methods are the supervised classification [6], non-supervised classification, object-oriented [7]–[8], neural networks [9], and decision tree classifications [10]. These methods have some disadvantages, such as multiple people participation, low efficiency, and insensitivity to changes in image quality [11]; therefore, they are not suitable for evaluation of differences in image quality.

The machine learning identification method based on Haar features was chosen to identify target objects. Firstly, Haar features used for object recognition were computed by inputting positive and negative samples, then the Adaboost algorithm was used to train the classifier, and the well-trained classifier was then used to analyze the tested images and automatically identify marked targets in RS images [12]. The machine learning identification method was characterized by advantages such as little human intervention, fast detection speed, and sensitivity to changes in image quality. Thus, this method is suitable to reflect the changes in RS data quality.

### (5) Sample Selection

The samples needed for training classifiers are intercepted from the tested images and divided into positive and negative samples. The former are the objects to be identified, i.e., the motor vehicles in the image, whereas the latter are other objects in the image that may interfere with motor vehicle identification, such as zebra crossings, bridges, shrubs, street lights, and boats in the rivers. In theory, the increase in the number of samples can improve the accuracy of the classifiers. In fact, when the number of samples is sufficiently large, the effect of improvement in classifiers is not obvious. After many comparative experiments, 500 positive samples with a pixel bitmap of the size of 32×32 and 1500 negative samples with pixel bitmap of sizes of 45×45 to 60×60 were selected for each image in this study.

### (6) Classifier Training

Applying the classifier training function loads positive and negative samples and calculates their Haar-like feature values. Then, applying the Adaboost algorithm trains different weak classifiers for the training set, and combining these generated weak classifiers with weights forms a stronger final classifier [13]–[14]. At the end of training, classifiers A, B, C, D, and E in the XML format corresponding to images (a), (b), (c), (d), and (e), respectively, are finally obtained.

### (7) Object Recognition and Mark

**Figure 3:**
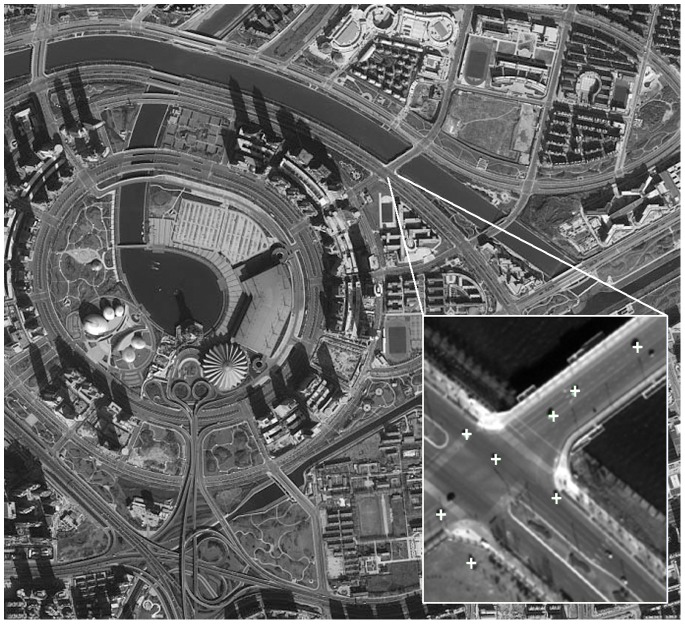
Object recognition and marking effect.

The vehicle recognition program written based on Microsoft Visual C++2008 and OpenCV 2.1 can directly call the previously trained classifiers A, B, C, D, and E to check the corresponding images. The search window in the recognition program is set to move for searching in the image. In order to monitor an object of unknown size in the image, it is necessary for the scanner using the search window with different size proportions to scan the picture several times and determine the parameters of the optimal search window. At the same time, a marker function is created and it will use the plus sign, “+,” to mark the searched vehicle, call the cvNamedWindow function to name the image window, and call the cvShowImage function to show the resultant image examined by the classifier. The result of object recognition is shown in Fig. 3.

### (8) Statistics of ORR

As the number of automobiles in the entire image is too large to accumulate statistical data of the recognition rate, 10 regions of fixed size in the image are randomly selected, and their recognition rate and average is calculated as the vehicle recognition rate in the image. When areas where a car contrasting with the background, such as streets and squares, are selected, the correctness of the program recognition can be accurately judged. The object recognition rate (ORR) is defined as:
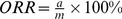
(1)where *a* is the number of vehicles correctly recognized, and *m* is the actual number of vehicles in the image.

### (9) Calculation of Image Quality Parameters

We selected a total of seven common image quality evaluation indexes, including the variance (d), kurtosis (K), information entropy (H), contrast (FC), sharpness (EAV), average gradient (AG), and edge intensity (EI), and wrote a MATLAB program to analyze five images: (a), (b), (c), (d) and (e). Table 1 lists the various parameter formulas and their corresponding explanations.

**Table 1:** Image quality parameters and their explanations.

Name	Expression and explanation
variance (d)	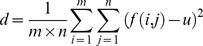 where m and n are the numbers of rows and columns in the image, respectively; f(i, j) is the grayscale value at point (i, j) in the image; u is the mean grayscale value in the image.
kurtosis (K)	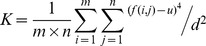 where m and n are the the numbers of rows and columns in the image, respectively; f(i, j) is the grayscale value at point (i, j) in the image; u is the mean grayscale value in the image; d is the variance.
entropy (H)	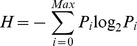 where Pi is the probability of a pixel grayscale value of i; max is the max grayscale value of a pixel.
contrast (FC)	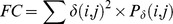 *_δ (i, j)_* is the grayscale difference between adjacent pixels; P(i, j) is the probability of pixel distribution of the grayscale difference between adjacent pixels δ.
sharpness (EAV)	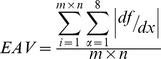 where m and n are the numbers of rows and columns in the image, respectively; α is the number of adjacent pixels; df/dx is the gradient between adjacent pixels.
average gradient (AG)	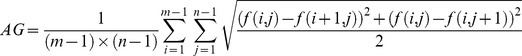 where m and n are the numbers of rows and columns in the image, respectively; f(i, j) is the grayscale value of the image at point (i, j).
edge intensity (EI)	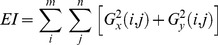 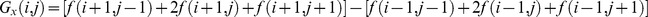 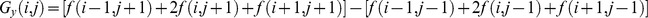 where m and n are the numbers of rows and columns in the image, respectively; f(i, j) is the grayscale value of the image at point (i, j).

## Conclusions

### (1) Analysis of ORR and Image Quality Parameters

Table 2 is obtained by comparing the image quality parameters of the original image and the degraded images with the vehicle recognition rates of corresponding images.

**Table 2:** Statistics of ORR and image quality parameters.

ImageNo.	ORR(%)	Variance(d)	Kurtosis(K)	Entropy(SH)	Sharpness(EAV)	Contrast(FC)	Averagegradient(AG)	Edgeintensity(EI)
(a)	81.95	1864.50	5.20	6.86	11.24	29.38	0.10	75.36
(b)	65.52	2393.39	4.46	6.79	20.72	34.26	0.16	85.82
(c)	64.58	1936.22	4.81	7.13	28.47	30.34	0.13	92.83
(d)	71.21	1651.02	4.39	6.86	5.37	28.41	0.07	61.34
(e)	73.11	1310.55	6.85	6.45	9.22	23.20	0.09	60.90

Nine commonly used indexes chosen in this study reveal the differences between images from the angles of the overall pixel grayscale distribution, the information entropy, and the edge sharpness. After processing, the image quality is significantly reduced, but the changes in these index values are unable to better show the changes in the image quality. Images obtained by different remote sensors have different resolutions. Those indexes displaying the characteristics of the histograms of images and the image sharpness cannot intuitively show the decline in the image quality. For images obtained by the same sensor at different conditions, their resolutions are equivalent. For example, after adding Gaussian noise, all the indexes except the recognition rate of image (c) cannot intuitively reflect the decline in image quality.

### (1) Assessment of Image Quality based on ORR

The vehicle identification rate in the image can better reflect the differences in the image quality; thus, if the rate acquired in the optimum condition is selected as a standard, the recognition rates of various images can be standardized, and the quality differences among different images can be compared. Because Worldview-2 satellite data are among the best commercial satellite data used in this study, the vehicle identification rate of the original image is 81.95%, close to the results of other studies [12]. Therefore, the recognition rate of 82% can be thought of as the recognition rate in the optimum condition. The ratios of the recognition rates of images (b), (c), (d), and (e) to the optimum recognition rates are 79.90%, 78.76%, 86.84%, and 89.16%.

As shown in Figure 1, image quality declines significantly after quality-degradation treatments, and the difficulty of object identification and information extraction increases. There are two vehicles labeled with 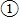 and 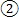. By a general visual comparison, we can observe that vehicles in (d) and (e) are easier to recognize than those in (b) and (c). Further analysis shows that vehicle 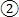 is better in (e) than in (d) and better in (c) than in (b). Therefore, by subjective visual perception, image quality in descending order is (a)>(e)>(d)>(c)>(b). This result is consistent with the standardized ORR data. Therefore, standardized ORR data can evaluate image quality more accurately than conventional digital image assessment indexes.

## Discussion

This study proposes to conduct target object recognition on Haar-like features of typical objects by a machine learning algorithm and evaluate image quality by identifying effects. Theoretically, when ORSI resolution is low, the image is fuzzy, or image noise is significant, Haar-like features of typical objects will be unobvious and unstable, which will reduce the effectiveness of the classifier, resulting in a lower target recognition rate.

Haar-like features reflect the grayscale features in the image region. They are capable of showing the structural relationship among adjacent pixels in the image and are very sensitive to a simple graphical structure, such as an edge or segment, in a particular direction (horizontal, vertical, or diagonal). The capacity for object recognition based on Haar-like features in nature has more to do with whether the image structural characteristics of the same object in different images are obvious and have a high degree of consistency. Lowering resolution, blurring, and other techniques can weaken the structural features of the same object in the image, whereas noise can decrease the consistency of the structural features of the same object in the image, and both will cause the training difficulty in the classifier to increase and the object recognition rate to decrease. This is similar to the image quality assessment method based on structural similarity. This is a full-reference assessment method and must define a reference image and compare image structures at the same location, thus greatly limiting its applications in the RS field. The use of object recognition based on Haar-like features for image quality assessment resolves this problem well because this method is used to detect structural deformation based on both significance and consistency of the image structural characteristics of the same type of objects.

In the field of remote sensing research and application, there is not a quantitative quality-evaluating method consistent with ORSI application effects. There will be many problems if quality indicators of digital image processing are used directly. In a new perspective, this study ensures maximum objectivity of evaluation by a machine learning algorithm and classifier training on one hand and conducts target object recognition by Haar-like features of typical objects on the other hand. Actually, this study examines whether the grayscale features in the image region are obvious and stable, which has a strong relationship with the ORSI application effects and reflects data quality. As a preliminary exploration and verification, this study uses only one type of optical remote sensing image (WorldView-2 panchromatic band) and only analyzes one type of object (vehicle). Therefore, the applicability of this evaluation method may be restricted, e.g., images with no vehicles. However, this study preliminarily validates that by using a machine learning algorithm with grayscale features in the image region, the quality of remote sensing data can be reflected by object recognition effects quantitatively and objectively. This is a new idea regarding quantitative evaluation of ORSI quality, and it is theoretically feasible to create a new ORSI evaluation system for different applications through this method. In practice, we can learn from quantitative remote sensing research methods to set up a large number of targets on the ground, then collect data and conduct target recognition. Thus, under a uniform standard, we can conduct quality evaluation on different data acquired by different sensors.

Additionally, our study was only focused on an experiment with panchromatic band data, and does not refer to the assessment of multispectral data. The latter may consider the analysis of the principal components of multiple wave bands and extract them for evaluation. With image analysis technology based on machine learning continuously developing, a more comprehensive, objective, and quantitative ORSI assessment systems fit to different mission requirements can be established.
